# Surgical Site Infections Are Associated With Higher Blood Loss and Open Access in General Thoracic Practice

**DOI:** 10.3389/fsurg.2021.656249

**Published:** 2021-06-25

**Authors:** Pauline Aeschbacher, Thanh-Long Nguyen, Patrick Dorn, Gregor Jan Kocher, Jon Andri Lutz

**Affiliations:** ^1^Department of Visceral Surgery and Medicine, Inselspital, Bern University Hospital, University of Bern, Bern, Switzerland; ^2^Department of Thoracic Surgery, Inselspital, Bern University Hospital, University of Bern, Bern, Switzerland

**Keywords:** surgical site infection, minimal invasive surgery, video-assisted thoracic surgery, thoracic surgery, complication

## Abstract

**Background:** Surgical site infections (SSIs) are the most costly and second most frequent healthcare-associated infections in the Western world. They are responsible for higher postoperative mortality and morbidity rates and longer hospital stays. The aim of this study is to analyze which factors are associated with SSI in a modern general thoracic practice.

**Methods:** Data were collected from our department's quality database. Consecutive patients operated between January 2014 and December 2018 were included in this retrospective study.

**Results:** A total of 2430 procedures were included. SSIs were reported in 37 cases (1.5%). The majority of operations were video-assisted (64.6%). We observed a shift toward video-assisted thoracic surgery in the subgroup of anatomical resections during the study period (2014: 26.7%, 2018: 69.3%). The multivariate regression analysis showed that blood loss >100 ml (*p* = 0.029, HR 2.70) and open surgery (*p* = 0.032, HR 2.37) are independent risk factors for SSI. The latter was higher in open surgery than in video-assisted thoracic procedures (*p* < 0.001). In the subgroup of anatomical resection, we found the same correlation (*p* = 0.043). SSIs are also associated with significantly longer mean hospital stays (17.7 vs. 7.8 days, *p* < 0.001).

**Conclusion:** As SSIs represent higher postoperative morbidity and costs, efforts should be made to maintain their rate as low as possible. In terms of prevention of SSIs, video-assisted thoracic surgery should be favored over open surgery whenever possible.

## Introduction

Surgical site infections (SSIs) are the second most frequent healthcare-associated infections in the United States and Europe (1). The overall incidence of SSI in general surgery was reported to be between 1.9 and 5.4% (2–4). Higher SSI rates can be found after colectomies (18.4%) (5). In thoracic surgery, there are some studies reporting SSI occurrence ranging from 0.3 to 6.1%, but no study looks specifically for factors associated with this burden in a recent period and in a fully implemented minimal invasive practice (6–11).

In the United States, SSIs represent the most costly healthcare-associated infection. They cause higher postoperative mortality, morbidity, and longer hospital stays (12). It is estimated that 55% of SSIs are preventable with the implementation of official recommendations (13, 14).

SSIs are defined by the United States Centers for Disease Control and Prevention (CDC) as “an infection that occurs after surgery in the part of the body where the surgery took place” (15). Superficial SSIs involve only the skin and subcutaneous tissue while deep SSIs affect tissues under the skin like muscle, fascia, adjacent organs/space opened, or manipulated during the operation or foreign body (15–18).

Many risk factors for SSI have been identified in general surgery, such as advanced age, previous radiation, previous skin and soft-tissue infection, high level of serum glucose, obesity, smoking, immunosuppression, malnutrition, malignant disease, hospitalization during the preoperative period, ongoing infection, blood transfusion, and longer operating time (13).

After a progressive introduction during the last three decades, video-assisted thoracic surgery (VATS) is commonly used. VATS in simple thoracic procedures such as pleural biopsy or pleurodesis is relatively easy to perform. VATS in more complex procedures such as lobectomy or segmentectomy can be a real challenge due to a longer learning curve. This results in longer operating times and higher intraoperative complication rates during that learning period (6). Its use in oncological procedures was a controversial subject until proven otherwise (8, 19–22). With experience, operation durations tend to be lower with less intraoperative complications (8, 19, 20). Recent studies report fewer postoperative complications, shorter hospital stay, and shorter chest tube duration without compromising oncological outcomes in VATS (8, 19, 23). Only two studies specifically analyze the risk factors for SSI in thoracic surgery in relation to open or minimally invasive surgery (10, 24). However, they are not able to demonstrate open surgery as a risk factor for SSI in comparison with minimal invasive surgery and they cover a long period of time before the implementation of VATS surgery for anatomical lung resections. Since VATS is now a well-established technique and is regularly performed with the development of expertise, we believe its positive influence on SSI rates in thoracic surgery can be better recognized.

Our study aims to assess the SSI rate over a 5-year period in a general thoracic “real-life” practice and to determine associated risk factors for SSI. During the same period, we implemented our VATS program for anatomical lung resection giving the possibility to measure its effect on this particular complication.

## Materials and Methods

### Patient Inclusion Criteria

Data were collected from the department quality database, which uses the tool of the Association for Quality Assurance in Surgery (AQC). Data are routinely entered prospectively into the database by a trained study nurse. Consecutive patients who underwent a thoracic operation between January 2014 and December 2018 at the Department of Thoracic Surgery of our university tertiary reference center were included. Tracheostomy, bronchoscopy, operation for vascular access, debridement of SSI, or reoperation for post-operative complication within 30 days after thoracic surgery was excluded. Patients <18 years of age were also excluded. This study was approved by the local ethic committee (Project-ID 2020-00850); each patient gave written consent for the use of his/her medical data during hospitalization.

### Patient Characteristics

The following clinicopathological variables were recorded: age, sex, BMI, ASA score, comorbidity, previous chemotherapy, or radiotherapy and chronic steroid use.

Patients' demographics includes also the following criteria: malignant and benign pathology of the main bronchus/trachea, of the mediastinum, primary lung malignancy, malignant pathology of the pleura, other malignant pathology, empyema, pneumothorax, pleural effusion and hemothorax, musculoskeletal pathology (unstable rib fracture, pectus excavatum/carinatum), complication after medical treatment or extra-thoracic surgery, and other benign pathology.

Subgroups were made according to operation type: simple thoracoscopy (biopsy, wedge resection, and pleurodesis), decortication and pleurectomy, lobectomy, segmentectomy, pneumonectomy, thymectomy, sympathectomy, mediastinum operation, and other.

For each operation, access type (open, VATS, and conversion) was specified, and whether it was elective or not. Operating time was reported in minutes, and estimated blood loss was reported in milliliters. A cutoff for blood loss was made at 100 ml, as literature reports higher cardiopulmonary complications in VATS surgery with blood loss above 100 ml (25).

Intraoperative complications were reported in four subgroups: vascular lesion, other organ lesions, operation interruption, and other intraoperative complications. Postoperative complications after the surgical procedure were classified according to the Clavien–Dindo classification (26). Clavien–Dindo grade ≥3b was considered a severe postoperative complication. Postoperative complications were divided into the following subgroups: respiratory complication (pneumonia, atelectasis, respiratory insufficiency, pneumothorax, and persistent air leakage, i.e., >5 days), cardiovascular complication, SSI, postoperative bleeding, and persistence of chest tube secretion (>200 ml/24 h for >7 days), bronchial stump insufficiency, hemothorax, chylothorax, empyema, and other complications. More than one complication can be reported per operation. Pneumonia, empyema, and bronchial stump insufficiency were not included in SSI but reported separately.

### SSI Definition

SSI was defined according to the abovementioned CDC definition (16). An SSI was diagnosed with the presence of redness, tenderness, heat, localized swelling, fever, purulent discharge, spontaneous wound dehiscence, and/or microorganism isolated form the wound fluid or tissue. The SSI was diagnosed through the attending surgeon.

### Surgical Procedure

In our clinic, VATS is performed using a two- or three-port access. For anatomical lung resections, we modified our approach to a uniportal VATS technique, consisting of a 3- to 5-cm incision length, in November 2014. No rib spreader was used for multiport VATS and soft wound protectors were used in uniportal VATS. Standard patient preparation for surgery includes disinfection with povidone-iodine solution (Betaseptic®). Prophylactic antibiotics were routinely administrated 30–45 min prior to skin incision with cefuroxime 1.5 g as standard dosage (or Clindamycin in case of β-lactam allergy). If an antibiotic treatment was already initiated, it was repeated prior to the incision in place of the standard prophylaxis. Antibiotics were adapted to the clinical situation, like the use of broader spectrum in case of empyema for example. For low infection risk surgery such as simple biopsy, the surgeon remains autonomous to decide whether a prophylaxis is needed or not. The skin was closed by means of a continuous intracutaneous suture with Monocryl®4–0.

During hospital stay, each patient was monitored for peri- and postoperative complications including SSI. A clinical nurse contacted each patient 7–10 days after hospital discharge by telephone to inquire about his/her general well-being and presence of any wound infection or discharge. Routine outpatient visits were performed 2–4 weeks after surgery.

### Endpoints

The endpoint of our study was to determine the risk factors associated with SSI. Since we initiated our uniportal VATS program during the study period, we also analyzed what effect the minimal invasive approach had on SSI, especially in anatomical lung resections.

### Statistical Analysis

Baseline characteristics are presented as medians (IQR) for continuous variables or frequencies for categorical variables. Aiming to identify factors associated with SSI, the following clinicopathological variables were analyzed using a univariate logistic regression model: previous chemotherapy or radiotherapy, chronic use of a corticosteroid, diabetes mellitus, comorbidity, ASA status, blood loss, access type, BMI, and log-transformed operation time in order to have it normally distributed. The maximal number of variable included in the multivariate analysis will be determined according to the incidence of SSI rate (according to the “one in ten rule”). In the subsequent multivariate analysis, the factors with significant *p*-value in univariate analysis were entered in a logistic regression model to identify predictors for SSI. Fisher's exact test was used to compare the overall number of SSIs by type of operation. Mann–Whitney *U* test and Kruskal–Wallis test were used to compare continuous variables and Fisher's exact test for categorical variables for comparing VATS vs. conversion vs. open surgery group. *p*-values < 0.05 were considered statistically significant. Analyses were done using Stata 15 (Stata, RRID:SCR_012763).

## Results

### Patient Characteristics

For the reviewed period, 2,671 patients were operated at our department. Due to the abovementioned exclusion criteria, 241 procedures were excluded, leaving 2,430 patients/operations fulfilling the inclusion criteria (2014: 431 patients, 2015: 454, 2016: 476, 2017: 509, 2018: 560). Clinicopathological characteristics are summarized in Table 1.

**Table 1:** Clinicopathological characteristics of 2,430 patients undergoing a thoracic surgery.

**Variable**	**All *n* (%) or median (IQR) *N* = 2,430**
Age	62.0 (49.0; 71.0)
Female	847 (34.9)
BMI (kg/m^2^)	24.5 (21.6; 27.8)
ASA score	
<3	838 (34.5)
≥3	1,577 (64.9)
Unknown	15 (0.6)
Risks factors, comorbidity	
Cardiovascular disease	462 (19.0)
Pulmonary disease	610 (25.1)
Neurological/psychiatric disease	189 (7.8)
Kidney disease	197 (8.1)
Liver disease	68 (2.8)
Diabetes mellitus	227 (9.3)
Oncological disease	1,170 (48.1)
No comorbidity	688 (28.3)
Previous chemotherapy	254 (10.5)
Previous radiotherapy	128 (5.3)
Chronic steroid use	65 (2.7)
Main diagnosis	
Main bronchus, trachea pathology (malignant/benign)	7 (0.3)/1 (0.04)
Primary lung malignancy (NSCLC)	690 (28.4)
Mediastinum pathology (malignant/benign)	31 (1.3)/56 (2.3)
Malignant pleural pathology (mesothelioma)	104 (4.4)
Malignant pathology other	329 (13.5)
Benign pathology other	493 (20.3)
Empyema	208 (8.6)
Pneumothorax	147 (6.0)
Pleural effusion/hemothorax	83 (3.4)/31 (1.3)
Operation on the musculoskeletal apparatus	188 (7.7)
Postoperative complication	62 (2.6)

*ASA, american society of anesthesiologists; BMI, body mass index; NSCLC, non-small cell lung carcinoma*.

### Perioperative Outcome

Elective surgery was mainly performed (93.4%). Antibiotics were administrated prior to the incision or were already prescribed as a therapy in 97.1% of the cases. The median operation time was 85 min. In 24.2% of operations, blood loss was >100 ml. Median hospitalization time was 6 days. For 104 (4.3%) operations, an intraoperative complication was reported. The global postoperative complication rate was 39.3% (*n* = 954), and there were 4.4% (*n* = 108) complications with a Clavien–Dindo grade ≥3b. Postoperative mortality was 0.7% (Table 2).

**Table 2:** Perioperative characteristics of 2,430 patients undergoing a thoracic surgery.

**Variable**	**All *n* (%) or median (IQR) *N* = 2,430**
Operation type	
Thoracoscopy	689 (28.4)
Decortication, pleurectomy	195 (8.0)
Lobectomy	363 (14.9)
Segmentectomy	285 (11.7)
Pneumonectomy	52 (2.1)
Thymectomy	70 (2.9)
Sympathectomy	57 (2.3)
Operation on the mediastinum	186 (7.7)
Other	533 (21.9)
Access type	
Open	683 (28.1)
Video-assisted-thoracoscopy	1,569 (64.6)
Conversion	50 (2.1)
Unknown	128 (5.3)
Antibiotic prophylaxis	
Prophylaxis prior to incision or therapeutic	2,359 (97.1)
Prophylaxis after incision	28 (1.2)
No antibiotics	37 (1.5)
Unknown	6 (0.2)
Elective operation	2,269 (93.4)
Operation time (min)	85.0 (50.0; 138.0)
Blood loss >100 ml	589 (24.2)
Hospitalization duration (days)	6.0 (4.0; 9.0)
Intraoperative complications	104 (4.3)
Vascular lesion	18 (0.7)
Lesion of other organ	6 (0.2)
Operation Interruption	6 (0.2)
Other	76 (3.1)
Postoperative complications (all Clavien–Dindo Grad)	954 (39.3)
Respiratory complication	121 (5.0)
Pneumothorax, persistent air leak	98 (4.0)
Cardiovascular complication	59 (2.4)
Surgical site infection	37 (1.5)
Bleeding	33 (1.4)
Decubitus	16 (0.7)
Persistence of drain secretion	10 (0.4)
Bronchial stump insufficiency	9 (0.4)
Hemothorax/chylothorax/empyema	5 (0.2)/6 (0.2)/8 (0.3)
Other	857 (35.2)
Clavien–Dindo classification ≥3b	108 (4.4)
Postoperative mortality (Grad 5)	17 (0.7)

### SSI

SSIs were reported in 37 cases (1.5%). For the logistic regression analysis, patients with unknown access type were excluded (128 patients). Univariate logistic regression analysis showed that ASA score ≥3, blood loss >100 ml, open surgery, and longer operation time were associated with higher SSI rate. For the access type, the open subgroup and the conversion subgroup were compared individually to the VATS subgroup. In the multivariate logistic regression analysis, blood loss >100 ml [*p* = 0.029, HR 2.70 CI (1.11–6.55)] and open surgery [*p* = 0.032, HR 2.37 CI (1.08–5.24)] were independent risk factors for SSI (Table 3). Conversion was not a risk factor for SSI. The only SSI occurring after a conversion was in a patient with severe comorbidities operated for an empyema.

**Table 3:** Analysis of factors associated with surgical site infection in 2,430 patients undergoing thoracic surgery.

**Variable**	**UV**		**MV**	
	**HR (95% CI)**	***p*-value**	**HR (95%-CI)**	***p*-value**
Previous radio- or chemotherapy	0.86 (0.30–2.44)	0.775		
Chronic steroid use	1.01 (0.14–7.49)	0.992		
Diabetes mellitus	1.53 (0.59–3.96)	0.384		
Comorbidity	2.06 (0.86–4.96)	0.101		
ASA score ≥3	2.30 (1.01–5.26)	**0.048**	1.54 (0.65–3.66)	0.328
Blood loss >100ml	4.21 (2.11–8.40)	** <0.001**	2.70 (1.11–6.55)	**0.029**
VATS	Ref.		Ref.	
Open	4.17 (2.10–8.28)	** <0.001**	2.37 (1.08–5.24)	**0.032**
Conversion	2.44 (0.31–19.05)	0.394		
BMI	1.00 (0.93–1.07)	0.985		
OP Time (min)	1.82 (1.10–3.01)	**0.02**	0.99 (0.53–1.82)	0.968

*ASA, american society of anesthesiologists; BMI, body mass index; CI, confidence interval; MV, multivariate logistic regression analysis; HR, Hazard ratio; UV, univariate logistic regression analysis; VATS, video-assisted thoracic surgery*.

**Figure 1:**
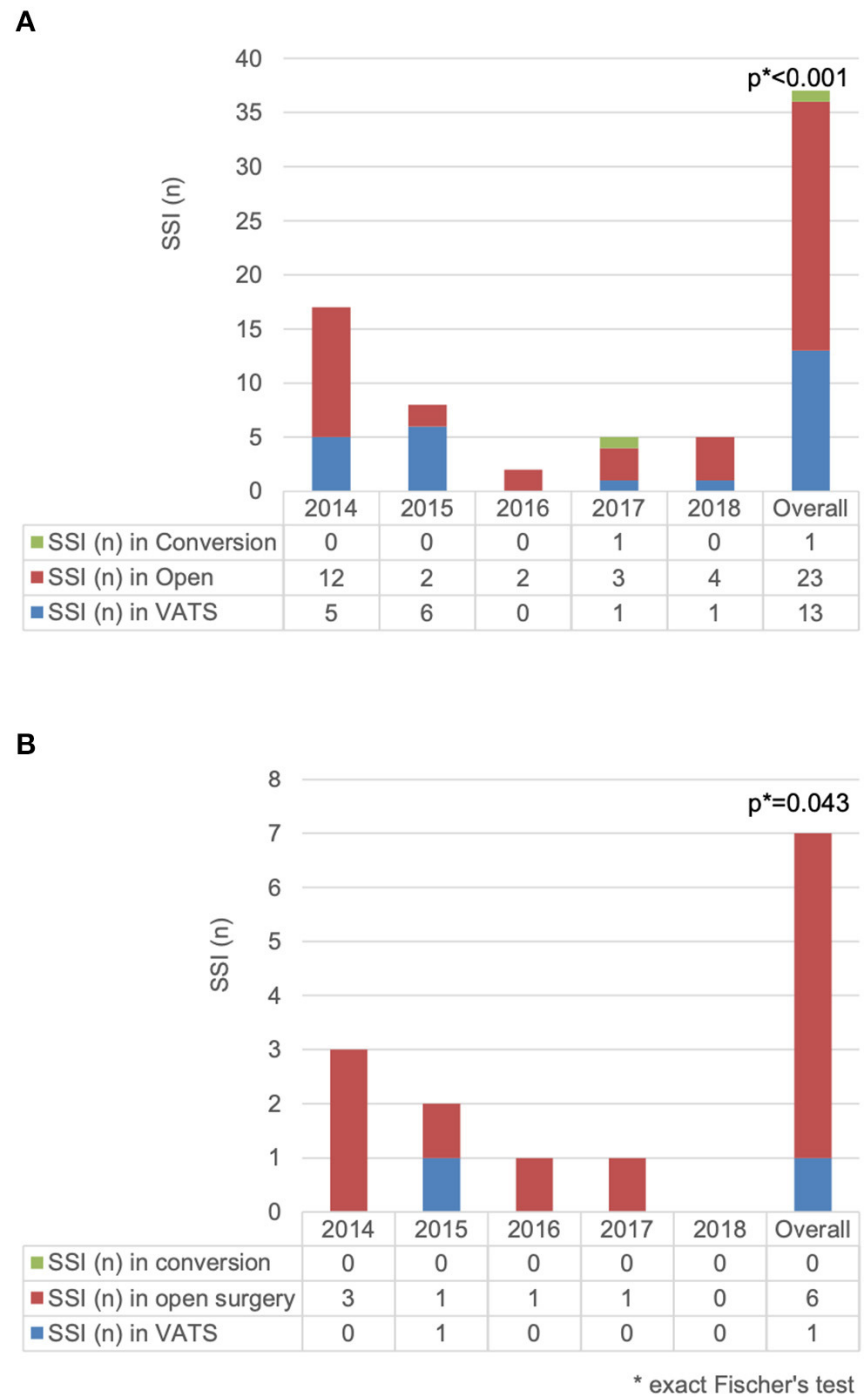
Comparison of surgical site infection (*n*) for **(A)** 2,430 patients undergoing thoracic surgery and **(B)** 700 patients undergoing anatomical resection according to access type.

The difference in SSI rate between open procedure and VATS was statistically significant [3.4 vs. 0.8% (*p* < 0.001)]. The difference was also significant in the anatomical resection group [2.3 vs. 0.2% (*p* = 0.043)] (Figure 1).

In the empyema subgroup (208 cases), the majority of cases were operated with a minimal invasive technique [VATS = 141 (67.8%) including 12 conversions vs. open = 59 (28.4%) and 8 missing data (3.8%)]. In this subgroup, 10 (4.8%) SSIs occurred (VATS = 3, open = 6, missing = 1).

SSIs were associated with longer hospital stays for all operations (6 vs. 14 days, *p* < 0.001) and for anatomical resections (7 vs. 17 days, *p* < 0.001) (Table 4). When SSIs were diagnosed, the mean hospital stay was 9.9 days longer for all thoracic operations and 14.5 days longer in anatomical resection. Persistent air leak was present in one patient with SSI, and even after exclusion of this patient, the length of stay was longer in the SSI subgroup (median 13.5 days). In the anatomical resection subgroup, the Number Needed to Treat using the VATS technique to prevent one SSI was 47.6.

**Table 4:** Comparison of hospitalization duration for 2,430 patients undergoing thoracic operation and for 700 patients undergoing anatomical resection according to SSI.

**Thoracic operation**	***N* = 2,430**	**Hospitalization duration (days) median (IQR)/mean**	*p*-value^**^
Without SSI	2,201	6 (4.0; 9.0)/7.8	** <0.001**
With SSI	37	14 (8.5; 21.0)/17.7	
Missing data	192		
Anatomical resection	*N* = 700		
Without SSI	693	7 (5.0; 9.5)/8.21	** <0.001**
With SSI	7	17 (16.0; 37.0)/22.71	

*IQR, interquartile range; SSI, surgical site infection*.

** *Mann–Whitney U test*.

### Open vs. VATS

**Figure 2:**
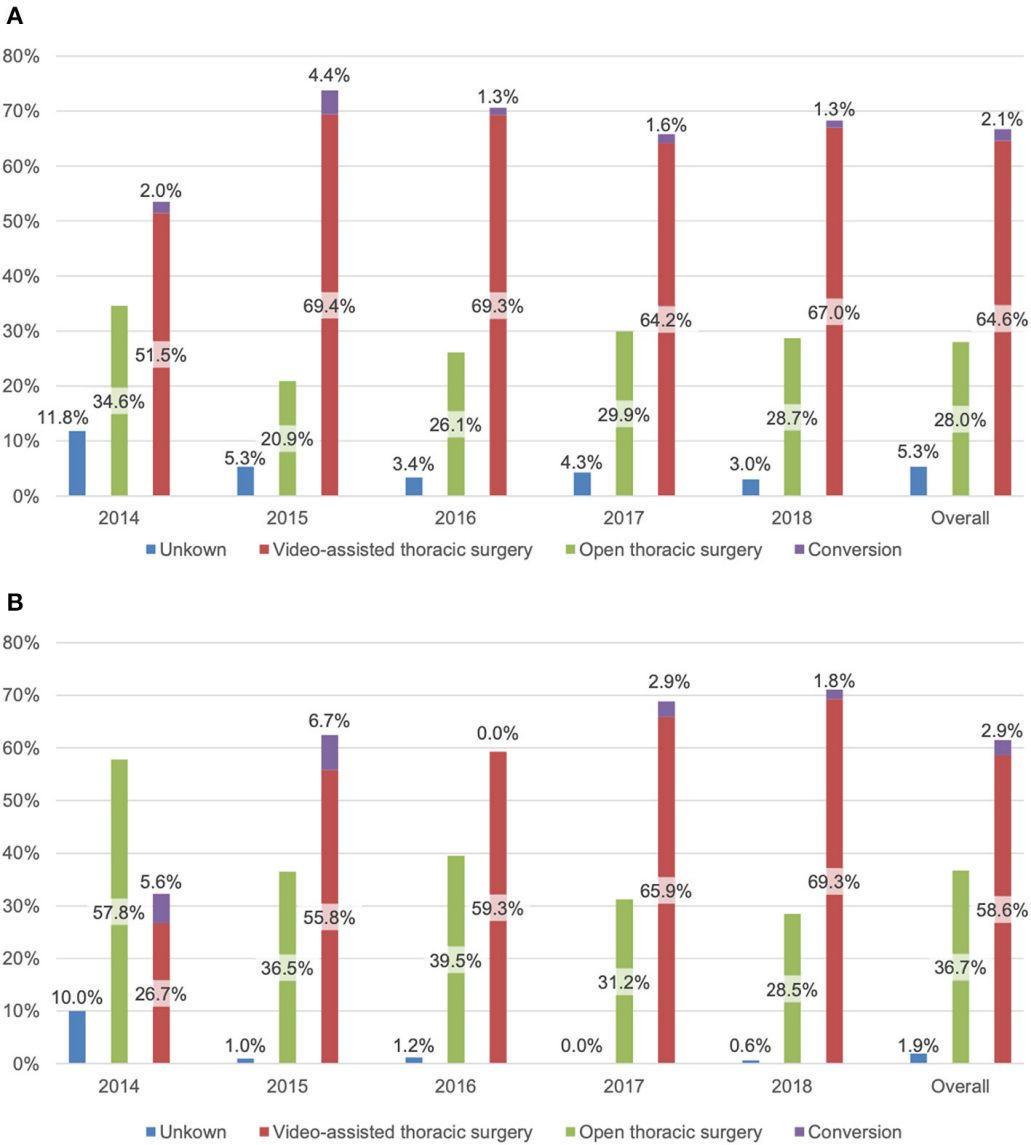
Access type (%) for **(A)** 2,430 patients undergoing thoracic surgery and **(B)** 700 patients undergoing anatomical resection.

The majority of operations were performed using a video-assisted approach (64.6%) with a conversion rate of 3.1% (50/1619). The proportion of VATS increased over the years, from 51.5% (*n* = 222) in 2014 to 67% (*n* = 375) in 2018. This is mainly due to the implementation of our VATS program for anatomical lung resections. The rate of conversions ranged from 1.8% (6/336) to 6.0% (20/335), with the highest rate in 2015 and the lowest rate in 2016 and 2018 (Figure 2A).

In the cohort undergoing an anatomical lung resection, 58.6% (*n* = 410) of procedures were done by VATS. In this subgroup, VATS surgery strongly increased over the years [26.7% (*n* = 24) to 69.3% (*n* = 115)] as it was progressively introduced at our department as a standard procedure. During the first 2 years (2014 and 2015), the conversion rate was higher [17.2% (5/29) and 10.8% (7/65), respectively], reflecting the learning curve in VATS anatomic resections (2016: 0%; 2017: 4.2%; 2018: 2.5%) (Figure 2B).

Table 5 shows the comparison of clinicopathological data between VATS (conversion included) and the open thoracic surgery group. Except for previous chemotherapy and operation indication, there was no statistical difference. However, the outcome differs with a lower intraoperative (*p* < 0.001) and postoperative morbidity (*p* < 0.001). Blood loss and operation time were also lower in the VATS group.

**Table 5:** Comparison of clinicopathological characteristics of 700 patients undergoing anatomical resection (VATS = 410, Open = 257, Conversion = 20, Missing data = 13) according to access type.

**Variable**	**VATS *n* (%) or median (IQR) *N* = 410**	**Conversion *n* (%) or median (IQR) *N* = 20**	**Open surgery *n* (%) or median (IQR) *N* = 257**	*p*-value^*^
Age	66 (59.0; 72.0)	64.5 (58.0; 69.75)	64 (57.0; 71.0)	0.063
Men	264 (64.4)	13 (65)	160 (62.3)	0.568
BMI	24.5 (21.70; 28.15)	24.2 (22.03; 28.2)	24.8 (21.9; 28.4)	0.551
ASA score ≥3	321 (78.3)	17 (85%)	212 (82.5)	0.237
Comorbidity				
Cardiovascular disease	104 (25.4)	4 (20%)	62 (24.1)	0.785
Pulmonary disease	138 (33.7)	6 (30)	95 (37.0)	0.364
Neuro/psych. disease	29 (7.1)	2 (10)	27 (10.5)	0.156
Kidney disease	34 (8.3)	0 (0)	18 (7.0)	0.766
Liver disease	14 (3.4)	0 (0)	7 (2.7)	0.821
Diabetes mellitus	35 (8.5)	1 (5)	29 (11.3)	0.226
Oncological disease	310 (75.6)	17 (85)	202 (78.6)	0.344
No comorbidity	34 (8.3)	1 (5)	20 (7.8)	0.886
Previous chemotherapy	41 (10.0)	3 (15)	51 (19.8)	**0.001**
Previous radiotherapy	31 (7.5)	3 (15)	21 (8.2)	0.886
Chronic steroid use	7 (1.7)	0 (0)	9 (3.5)	0.124
Operation indication				**0.001**
Oncological	348 (84.9)	20 (100)	238 (92.6)	
Benign	54 (13.2)	0 (0)	12 (4.7)	
Infectious	8 (2.0)	0 (0)	7 (2.7)	
Elective operation	409 (99.8)	20 (100)	253 (98.4)	0.068
Operation time (min)	122 (98.0; 152.0)	180 (139.5; 218.75)	174 (136.25; 224.75)	** <0.001**
Blood loss >100 ml	110 (26.8)	12 (60)	168 (65.4)	** <0.001**
Hospitalization duration (days)	6 (5.00; 8.00)	8 (6.25; 13.75)	9 (8.00; 12.00)	** <0.001**
Clavien–Dindo ≥3b	20 (4.9)	2 (10)	30 (11.7)	**0.003**
Postoperative mortality	1 (0.2)	0 (0)	4 (1.6)	0.068
Intraoperative complications	7 (1.7)	7 (35)	25 (9.7)	**0.001**
Postoperative complications	182 (44.4)	9 (45)	170 (66.1)	** <0.001**
Surgical site infection	1 (0.2)	0 (0)	6 (2.3)	**0.013**

*ASA, American society of anesthesiologists; BMI, body mass index; IQR, interquartile range VATS, video-assisted thoracic surgery*.

* *Fisher's exact test for categorical variables, Kruskal–Wallis test for continuous variables*.

## Discussion

Our study describes the SSI rate at our thoracic surgical department between 2014 and 2018. Open surgery and blood loss >100 ml were found to be independent risk factors for SSI in the multivariate analysis.

The overall SSI rate was 1.5%, which reflects current literature rates mentioned above (6–11). There is no consensus on criteria for the diagnosis of SSI (1, 4, 18). Up to 41 SSI definitions are reported in the literature. Only five were reported as standardized definitions: CDC-1988, CDC-1992, SISG (Surgical Infection Study Group), NPS (National Prevalence Survey), and PHLS (Public Health Laboratory Service) (1, 17, 27). This variety of SSI definitions can result in over-/underreporting of SSI rates (27).

We did not consider empyema and pneumonia as SSI, since both can be present in other situations (e.g., in case of bronchial stump insufficiency or atelectasis). Most studies reporting SSI in thoracic surgery did the same. As an exception, the second study of Imperatori et al. on the subject in 2017 includes pneumonia and empyema in SSI. This was not the case in their previous study in 2006 (9, 24).

Current WHO recommendations for the prevention of SSI are reported in “Global Guidelines for the Prevention of Surgical Site Infection” (1). Except for antimicrobial-coated sutures, our clinic puts effort into following the current recommendations for the prevention of SSI in a standardized way. Our strong adherence to current WHO recommendations probably played a role in the low SSI rate reported in our study.

We observed a good adherence to protocols for the administration of antibiotic prophylaxis (or the ongoing antibiotic treatment) in 97.1% of the cases. Antibiotic given after the incision (1.2%) can occur when collection of samples for microbial determination is required prior to the administration of antibiotics.

In colorectal surgery, the superiority of laparoscopic surgery has been demonstrated as well as its association with lower SSI rates (28). There are little data determining the influence of VATS on SSI rate especially for anatomical lung resection. Imperatori et al. reported a SSI incidence of 3.2% in 988 thoracic surgical procedures during the years 1996–2005. SSI rate was lower in wedge resections by VATS than in open surgery (5.5 vs. 17.9%, *p* < 0.001), but all anatomical resections were performed by an open approach (9). In his second study from 2006 to 2015, the multivariate analysis did not identify open surgery as a risk factor for SSI in comparison with VATS (24). Only 36 of the 512 anatomical resections were performed with VATS. Cvijanović et al. reported a SSI rate of 6.1% in thoracic surgery in 3,370 patients over a 12-year period (VATS: 2.14%, Open 7.1%), but the result was not significant in a multivariate analysis aiming to identify SSI risk factor and only 30 of the 1,319 anatomical resections were performed by VATS (10). In all these studies, the compared groups were rather heterogeneous, and although the study of Cvijanović et al. is one of the largest studies investigating this matter in thoracic surgery so far, the long study period includes a lot of changes in patient treatment (used disinfectant, preoperative antibiotics, surgical technique, etc.). Three studies based on large state or society databases mention SSI rate but not as one of their main endpoints. Falcoz et al. reported a significantly lower SSI rate in VATS lobectomy (0.2%) vs. open (0.7%, *p* = 0.0218) for primary non-small cell lung cancer in a propensity-matched analysis (21). Two other studies did not confirm the advantage of VATS lobectomy concerning the SSI rate (6, 23).

High blood loss is often a marker of longer and more difficult operations with probable need for transfusion and is associated with more complications, especially of cardiopulmonary origin, as well as shorter survival and immunity impairment (19, 25). Mean blood loss is reported to be lower in VATS surgery and could therefore indirectly influence the SSI rate in VATS operations (13, 19, 22). In our study, however, a blood loss >100 ml was an independent risk factor for SSI in the multivariate analysis.

In current literature, intraoperative complications tend to be identical or higher in VATS than in open surgery (6, 23). The length of hospital stay, overall hospitalization costs, and postoperative complication seem similar or even better for VATS (6, 8, 20). The implementation of the VATS program at our department in the initial study period showed typical characteristics of the learning curve with a conversion rate falling from 4.4% to 1.3–1.6%. Intraoperative and postoperative complications were lower in VATS along the whole period. Cvijanovic et al. as well as our study showed that conversion is not associated with a higher SSI rate (*p* = 0.733) (10). It seems therefore reasonable to attempt a minimally invasive operation even when the risk of conversion is high.

As SSIs represent longer hospital stay, rehospitalization, reoperation, and specific care, it is associated with higher costs. Cost burden associated with SSI in Europe is estimated at 1.47–19.1 billion Euro with 7–14 days of extended hospital stay (4, 29). Our study reports a longer hospital stay in the SSI subgroup. However, other postoperative complications were also more frequent in this subgroup. Therefore, the extended hospitalization can also be explained with a complication cascade including SSI and not solely resulting from an SSI occurrence.

Our study presents the limitation of being retrospective, and the comparison of VATS and open surgery groups for anatomical resection has an inherent bias. There were more preoperative chemotherapies (10.2 vs. 19%) and indication for operation was more often oncological in the open group (92.6 vs. 84.9%). Patient requiring neoadjuvant chemotherapy often have larger tumors and positive mediastinal lymph nodes and, consequently, open surgery was preferred, especially during the learning phase. However, it is important to notice that the rate of patients operated for infectious diseases (suspicion of malignancy for nodules of organizing pneumonitis) were similar in the open and VATS subgroups (2.7% resp. 2.0%). Interestingly, the 20 cases necessitating a conversion had no SSI. During the learning phase, complex anatomical resection was performed with an open access and could be responsible for a higher rate of intraoperative complications and blood loss. However, this can also be the case for the VATS subgroup where operations performed during the learning phase were included.

We deliberately included cases of empyema in our study for a real-life global overview on the causes of SSI. The majority of those cases were operated with a minimally invasive technique and cannot be an explanation for the global lower SSI rate in the VATS subgroup. Nevertheless, our study is one of the largest study to investigate the matter of SSI in general thoracic surgery practice and especially includes the most homogeneous patients and standardized procedures so far.

## Conclusion

SSI is associated with open approach and higher blood loss in a general thoracic surgery practice. They are also correlated to higher postoperative morbidity and longer hospital stay, leading to higher overall costs, and attempts should be made to prevent them. From the two risk factors identified, only the minimal invasive approach can be influenced by the surgeon, and since conversion does not seem to harm the patient, we advocate a primary VATS approach whenever possible.

## Data Availability Statement

The original contributions presented in the study are included in the article/supplementary material, further inquiries can be directed to the corresponding author/s.

## Ethics Statement

The studies involving human participants were reviewed and approved by Cantonal Ethics Committee of Bern. The patients/participants provided their written informed consent to participate in this study.

## Author Contributions

PA, JAL, and GJK: conception and design. T-LN, PD, and JAL: administrative support. T-LN, PD, GJK, and JAL: provision of study materials or patients. PA and JAL: collection and assembly of data and data analysis and interpretation. All authors contributed to the article and approved the submitted version.

## Conflict of Interest

The authors declare that the research was conducted in the absence of any commercial or financial relationships that could be construed as a potential conflict of interest.

## References

[1] Allegranzi B Bischoff P Kubilay Z de Jonge S Zayed B Abbas M. Global Guidelines For the Prevention of Surgical Site Infection. Geneva: World Health Organization (2018).

[2] Sax H Uckay I Balmelli C Bernasconi E Boubaker K Muhlemann K. Overall burden of healthcare-associated infections among surgical patients. Results of a national study. Ann Surg. (2011) 253:365–70. 10.1097/SLA.0b013e318202fda9 21217517

[3] Mu Y Edwards JR Horan TC Berrios-Torres SI Fridkin SK. Improving risk-adjusted measures of surgical site infection for the national healthcare safety network. Infect Control Hosp Epidemiol. (2011) 32:970–86. 10.1086/662016 21931247

[4] Leaper DJ van Goor H Reilly J Petrosillo N Geiss HK Torres AJ. Surgical site infection - a European perspective of incidence and economic burden. Int Wound J. (2004) 1:247–73. 10.1111/j.1742-4801.2004.00067.x 16722874 PMC7951634

[5] Staszewicz W Eisenring MC Bettschart V Harbarth S Troillet N. Thirteen years of surgical site infection surveillance in Swiss hospitals. J Hosp Infect. (2014) 88:40–7. 10.1016/j.jhin.2014.06.003 25063012

[6] Gopaldas RR Bakaeen FG Dao TK Walsh GL Swisher SG Chu D. Video-assisted thoracoscopic versus open thoracotomy lobectomy in a cohort of 13,619 patients. Ann Thorac Surg. (2010) 89:1563–70. 10.1016/j.athoracsur.2010.02.026 20417778

[7] Turna A Kutlu CA Ozalp T Karamustafaoglu A Mulazimoglu L Bedirhan MA. Antibiotic prophylaxis in elective thoracic surgery: cefuroxime versus cefepime. Thorac Cardiovasc Surg. (2003) 51:84–8. 10.1055/s-2003-38991 12730816

[8] Laursen LO Petersen RH Hansen HJ Jensen TK Ravn J Konge L. Video-assisted thoracoscopic surgery lobectomy for lung cancer is associated with a lower 30-day morbidity compared with lobectomy by thoracotomy. Eur J Cardiothorac Surg. (2016) 49:870–5. 10.1093/ejcts/ezv205 26088592

[9] Imperatori A Rovera F Rotolo N Nardecchia E Conti V Dominioni L. Prospective study of infection risk factors in 988 lung resections. Surg Infect. (2006) 7(Suppl. 2):S57–60. 10.1089/sur.2006.7.s2-57 16895508

[10] Cvijanovic VS Ristanovic AS Maric NT Vesovic NV Kostovski VV Djenic LV. Surgical site infection incidence and risk factors in thoracic surgical procedures: a 12-year prospective cohort study. J Infect Dev Ctries. (2019) 13:212–18. 10.3855/jidc.11240 32040450

[11] Boffa DJ Allen MS Grab JD Gaissert HA Harpole DH Wright CD. Data from the society of thoracic surgeons general thoracic surgery database: the surgical management of primary lung tumors. J Thorac Cardiovasc Surg. (2008) 135:247–54. 10.1016/j.jtcvs.2007.07.060 18242243

[12] Anderson DJ Podgorny K Berrios-Torres SI Bratzler DW Dellinger EP Greene L. Strategies to prevent surgical site infections in acute care hospitals: 2014 update. Infect Control Hosp Epidemiol. (2014) 35:605–27. 10.1086/676022 24799638 PMC4267723

[13] Zimlichman E Henderson D Tamir O Franz C Song P Yamin CK. Health care-associated infections: a meta-analysis of costs and financial impact on the US health care system. JAMA Intern Med. (2013) 173:2039–46. 10.1001/jamainternmed.2013.9763 23999949

[14] Umscheid CA Mitchell MD Doshi JA Agarwal R Williams K Brennan PJ. Estimating the proportion of healthcare-associated infections that are reasonably preventable and the related mortality and costs. Infect Control Hosp Epidemiol. (2011) 32:101–14. 10.1086/657912 21460463

[15] United States Centers for Disease Control and Prevention. Available online at: https://www.cdc.gov/HAI/ssi/ssi.html (accessed January 13, 2021).

[16] Horan TC Gaynes RP Martone WJ Jarvis WR Emori TG. CDC definitions of nosocomial surgical site infections, 1992: a modification of CDC definitions of surgical wound infections. Infect Control Hosp Epidemiol. (1992) 13:606–8. 10.2307/30148464 1334988

[17] Bruce J Russell EM Mollison J Krukowski ZH. The measurement and monitoring of surgical adverse events. Health Technol Assess. (2001) 5:1–94. 10.3310/hta5220 11532239

[18] Karapinar K Kocaturk CI. The effectiveness of sterile wound drapes in the prevention of surgical site infection in thoracic surgery. Biomed Res Int. (2019) 2019:1438793. 10.1155/2019/1438793 30886857 PMC6388313

[19] Wang Z Pang L Tang J Cheng J Chen N Zhou J. Video-assisted thoracoscopic surgery versus muscle-sparing thoracotomy for non-small cell lung cancer: a systematic review and meta-analysis. BMC Surg. (2019) 19:144. 10.1186/s12893-019-0618-1 31615490 PMC6794906

[20] Paul S Isaacs AJ Treasure T Altorki NK Sedrakyan A. Long term survival with thoracoscopic versus open lobectomy: propensity matched comparative analysis using SEER-Medicare database. BMJ. (2014) 349:g5575. 10.1136/bmj.g5575 25277994 PMC4183188

[21] Falcoz PE Puyraveau M Thomas PA Decaluwe H Hurtgen M Petersen RH. Video-assisted thoracoscopic surgery versus open lobectomy for primary non-small-cell lung cancer: a propensity-matched analysis of outcome from the European society of thoracic surgeon database. Eur J Cardiothorac Surg. (2016) 49:602–9. 10.1093/ejcts/ezv154 25913824

[22] Yang CJ Kumar A Klapper JA Hartwig MG Tong BC Harpole Jr DH. A national analysis of long-term survival following thoracoscopic Vs. open lobectomy for stage I non-small-cell lung cancer. Ann Surg. (2019) 269:163–71. 10.1097/SLA.0000000000002342 28799982

[23] Paul S Sedrakyan A Chiu YL Nasar A Port JL Lee PC. Outcomes after lobectomy using thoracoscopy vs thoracotomy: a comparative effectiveness analysis utilizing the nationwide inpatient sample database. Eur J Cardiothorac Surg. (2013) 43:813–7. 10.1093/ejcts/ezs428 22826474

[24] Imperatori A Nardecchia E Dominioni L Sambucci D Spampatti S Feliciotti G. Surgical site infections after lung resection: a prospective study of risk factors in 1,091 consecutive patients. J Thorac Dis. (2017) 9:3222–31. 10.21037/jtd.2017.08.122 29221299 PMC5708450

[25] Li S Zhou K Lai Y Shen C Wu Y Che G. Estimated intraoperative blood loss correlates with postoperative cardiopulmonary complications and length of stay in patients undergoing video-assisted thoracoscopic lung cancer lobectomy: a retrospective cohort study. BMC Surg. (2018) 18:29. 10.1186/s12893-018-0360-0 29792183 PMC5966911

[26] Dindo D Demartines N Clavien PA. Classification of surgical complications: a new proposal with evaluation in a cohort of 6336 patients and results of a survey. Ann Surg. (2004) 240:205–13. 10.1097/01.sla.0000133083.54934.ae 15273542 PMC1360123

[27] Wilson AP Treasure T Sturridge MF Gruneberg RN. A scoring method (ASEPSIS) for postoperative wound infections for use in clinical trials of antibiotic prophylaxis. Lancet. (1986) 1:311–3. 10.1016/S0140-6736(86)90838-X 2868173

[28] Kagawa Y Yamada D Yamasaki M Miyamoto A Mizushima T Yamabe K. The association between the increased performance of laparoscopic colon surgery and a reduced risk q8 surgical site infection. Surg Today. (2019) 49:474–81. 10.1007/s00595-019-1760-1 30684051 PMC6526142

[29] Jenks PJ Laurent M McQuarry S Watkins R. Clinical and economic burden of surgical site infection (SSI) and predicted financial consequences of elimination of SSI from an English hospital. J Hosp Infect. (2014) 86:24–33. 10.1016/j.jhin.2013.09.012 24268456

